# The roles of several sensory neurons and the feedback from egg laying in regulating the germline response to a sex pheromone in *C. elegans* hermaphrodites

**DOI:** 10.17912/micropub.biology.000523

**Published:** 2022-02-02

**Authors:** Erin Z. Aprison, Ilya Ruvinsky

**Affiliations:** 1 Department of Molecular Biosciences, Northwestern University

## Abstract

Animals broadcast small molecule pheromones that can alter behavior and physiology in conspecifics. Neuronal circuits that regulate these processes remain largely unknown. In *C. elegans*, male-enriched ascaroside sex pheromone ascr#10, in addition to behavioral effects, expands the population of germline precursor cells in hermaphrodites. Previously, we identified several sensory neurons required for this effect. We also found that feedback from egg laying acts via serotonergic signaling to license the pheromone response in reproducing adults. Here, using newly available reagents, we confirm and extend several of our previous conclusions: a) the ADL neurons are essential for the ascr#10 response, b) phasmid neurons (PHA and PHB) are unlikely to be involved in the ascr#10 response, c) the *mod-1* receptor is the main conduit of the serotonergic feedback from egg laying, and d) serotonin remains the only currently known signal of this feedback. Our findings better define the neuronal circuits that mediate the germline response to the major male pheromone.

**Figure 1. Effects of several mutations affecting neuronal functions on the response of hermaphrodite germline to male pheromone ascr#10:**
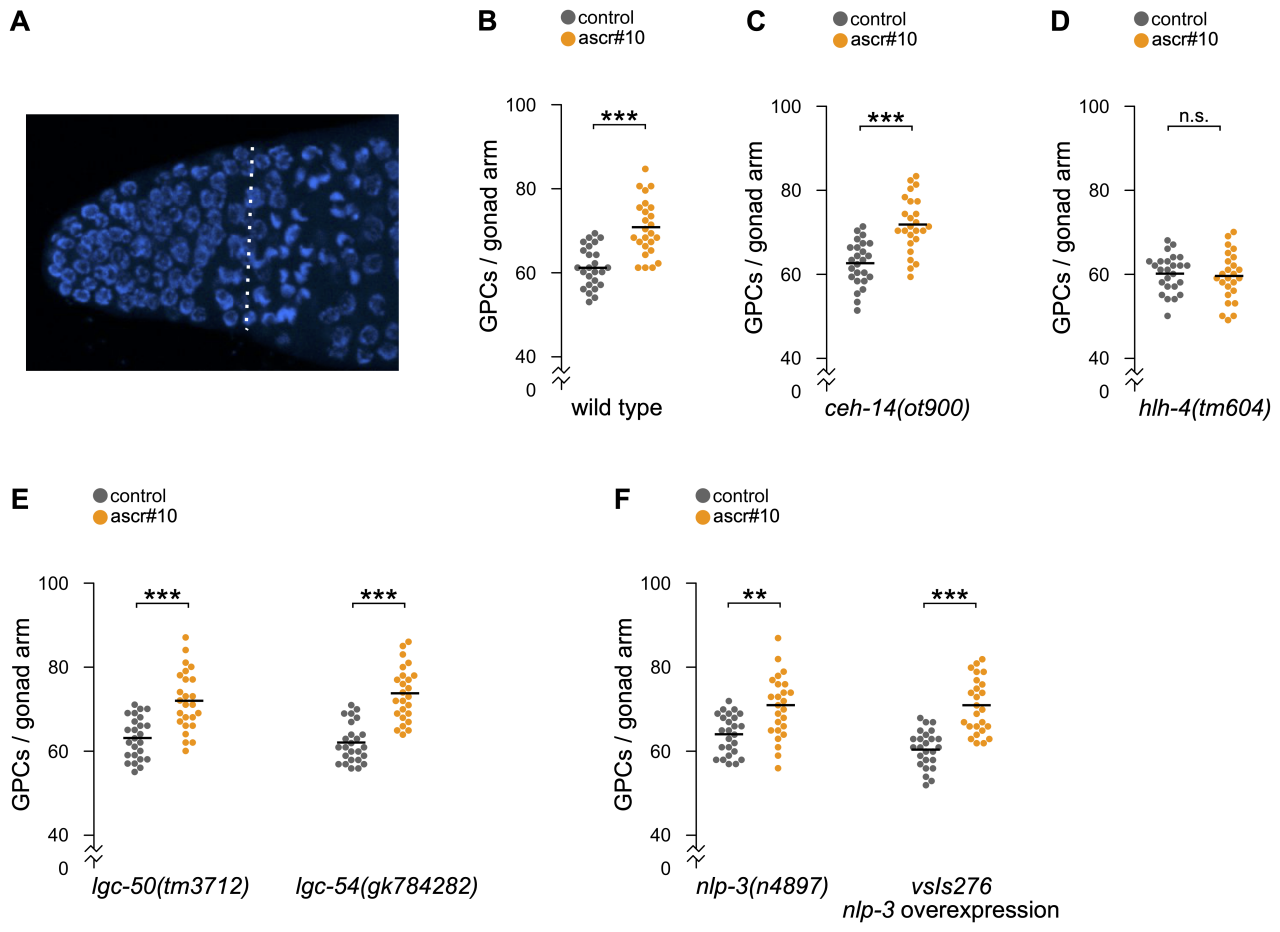
(A) Representative image of a DAPI-stained distal germline of a 5-day old hermaphrodite. Distal to the left, proximal to the right. Nuclei to the left of the vertical dotted line were counted as the germline precursor cell (GPC) population. (B) Wild type N2 response to ascr#10 in the germline. In this, as in all subsequent panels, each dot denotes the number of GPCs in one gonad arm of one hermaphrodite; grey dots represent animals on control plates, orange dots represent animals exposed to ascr#10 starting at 48 hours after release from L1 arrest (after L4/adult transition, but before onset of egg laying) until being tested on Day 5 of adulthood. (C) The germline response of *ceh-14(ot900)* to ascr#10. (D) The germline response of *hlh-4(tm604)* to ascr#10. (E) The germline response to ascr#10 of mutants in two recently identified serotonin receptors, *lgc-50(tm3712)* and *lgc-54(gk784282)*. (F) The germline response to ascr#10 of *nlp-3(n4897)* and *nlp-3* overexpressing strain (*vsIs276*). N=25 for all conditions. Asterisks indicate statistical significance as determined by the Kolmogorov-Smirnov test (** p<0.01, ***p<0.001).

## Description

The distal-most portion of the hermaphrodite germline in *C. elegans* is referred to as the Progenitor Zone (Figure 1A). Most cells in this population are mitotically-cycling germline precursors, but some have progressed as far as the early stages of prophase of meiosis I (Fox *et al.*, 2011). The size of the Progenitor Zone is sensitive to a variety of environmental factors (Hubbard and Schedl, 2019). For example, in the presence of physiological concentrations of ascr#10, the most abundant male-enriched excreted ascaroside (Izrayelit *et al.*, 2012), hermaphrodites have an enlarged population of germline precursor cells (Figure 1B; (Aprison and Ruvinsky, 2016)).

Previously, we identified several neurons involved in the germline response to ascr#10. Because loss-of-function mutations in *ocr-2* and *osm-9* genes eliminated the germline response, we focused on six pairs of sensory neurons – amphids ADF, ADL, ASH, and AWA and phasmids PHA and PHB – in which expression of these two genes overlaps (Aprison and Ruvinsky, 2017). A recent single cell RNAseq atlas supports the idea of a narrow overlap between expression patterns of *ocr-2* and *osm-9*, identifying PVD as the only additional neuronal type in the overlap (Taylor *et al.*, 2021). In the original study, we focused on amphid neurons because loss of a LIM homeobox gene *ceh-14* did not appear to alter the ascr#10 response (Aprison and Ruvinsky, 2017). The *ceh-14* gene is thought to be expressed in ~10 (Kagoshima *et al.*, 2013) to ~20 (Taylor *et al.*, 2021) neuronal types, including phasmid sensory neurons PHA and PHB. It was not clear whether the *ceh-14*(*ch3*) allele tested in our original study (Aprison and Ruvinsky, 2017), caused a complete and stable loss of function. More recently, a new mutant allele *ceh-14(ot900)*, a near complete deletion of the locus, became available (Bayer and Hobert, 2018). Hermaphrodites carrying this allele had the same germline response to ascr#10 as the wild type animals (Figure 1C), confirming our original conclusion that PHA and PHB neurons are dispensable for the germline response to ascr#10.

Of the four pairs of amphid neurons implicated by the *ocr-2*/*osm-9* overlap (ADF, ADL, ASH, and AWA), our original study focused on ADL because expression of the wild type OCR-2 protein in these, but not in the other three pairs, rescued the *ocr-2(lf)* inability to respond to ascr#10 (Aprison and Ruvinsky, 2017). To obtain additional evidence for the role of ADL neurons, we tested a loss-of-function mutation in the HLH-4 transcription factor that defines ADL identity (Masoudi *et al.*, 2018). This gene appears to be expressed exclusively in ADL neurons, as judged by a fosmid-based reporter transgene (Masoudi *et al.*, 2018) and the single cell RNAseq atlas (Taylor *et al.*, 2021). *hlh-4(tm604)* animals showed no increase of germline precursor cells in the presence of ascr#10 (Figure 1D), confirming our original conclusion that ADL neurons are required for the germline response to this male pheromone.

The ability of ascr#10 to enlarge the population of germline precursor cells is restricted to actively reproducing hermaphrodites because egg laying licenses pheromone response (Aprison and Ruvinsky, 2019b). The feedback of active reproduction involves the serotonin signal from the command motoneurons of egg laying, HSN (Aprison and Ruvinsky, 2019b). Previously, we tested mutants in six genes thought to act as serotonin receptors – *ser-1*, *ser-4*, *ser-5*, *ser-7*, *mod-1*, and *lgc-40* – and found that only one, the inhibitory serotonin-gated chloride channel *mod-1*, is essential for the increase of the number of germline precursors on ascr#10 (Aprison and Ruvinsky, 2019a). Since then, two additional serotonin receptors have been reported: *lgc-50* that appears to be exclusive for serotonin and *lgc-54* that also responds to dopamine and tyramine (Morud *et al.*, 2021). Hermaphrodites carrying mutations in either of these two genes did not discernibly differ from their wild type counterparts (Figure 1E), further supporting the idea that *mod-1* is the only currently annotated serotonin receptor required for the germline effects of ascr#10.

Command motoneurons HSN release serotonin along with neuropeptide NLP-3 to stimulate egg laying episodes (Brewer *et al.*, 2019). Because feedback from egg laying, mediated at least in part by serotonin, is required for increasing the number of germline precursors in the presence of ascr#10 (Aprison and Ruvinsky, 2019b), we tested whether NLP-3 plays a role in this feedback. We found that neither nlp-3 loss (nlp-3(n4897)), nor overexpression (nlp-3 (vsIs276)), noticeably affected germline response to ascr#10 (Figure 1F). These results leave serotonin as the only currently known mediator of the feedback from the egg-laying apparatus that limits the ascr#10 effects on the germline to actively reproducing animals.

## Methods

We used standard *C. elegans* methods as previously published (Aprison and Ruvinsky, 2017, 2019a, b). Briefly, worms were synchronized by hypochlorite treatment and subsequent overnight incubation in M9, after which the synchronized L1 larvae were placed (in groups of ~30) on agar plates seeded with *E. coli* OP50. 48 hours later, when worms were young, but pre-reproductive adults, they were transferred to OP50-seeded plates that were either control or conditioned with synthetic ascr#10 (gift of F. C. Schroeder). This protocol ensured that all experiments were paired with a control. On Day 5 of adulthood, hermaphrodites were stained with DAPI (4′,6-diamidino-2-phenylindole) as described (Aprison and Ruvinsky, 2016) and the germline precursor cells were counted. The boundary between the germline precursor population and the more proximal transition zone is defined by the appearance of crescent-shaped nuclei that have progressed to leptotene/zygotene stages of meiotic prophase (Crittenden *et al.*, 2006; Hansen *et al.*, 2004). Additional protocol details are available upon request.

## Reagents

**Table:** 

**Strain**	**Genotype**	**Obtained from**
N2	wild type	CGC
OH15422	*ceh-14(ot900)*	CGC
OH16755	*hlh-4(tm604)*	Hobert lab
AQ4347	*lgc-50(tm3712)*	Morud lab
AQ4493	*lgc-54(gk784282)*	Morud lab
LX2388	*nlp-3(n4897)*	Koelle lab
LX2518	*vsIs276* (*nlp-3* overexpression)	Koelle lab
